# Behavioral intervention for mental health of pregnant women experiencing domestic violence: a randomized controlled trial

**DOI:** 10.3389/fpubh.2025.1541169

**Published:** 2025-06-09

**Authors:** Meerambika Mahapatro, Sudeshna Roy, Poonam Nayar, Ashwini Jadhav, Suruchi Panchkaran, Divyanshu Srivastava, Sudha Prasad

**Affiliations:** ^1^National Institute of Health and Family Welfare, New Delhi, India; ^2^Maulana Azad Medical College, University of Delhi, New Delhi, India

**Keywords:** mental health, domestic violence, depression, quality of life, PTSD, RCT, anxiety, coping

## Abstract

**Background:**

The intersection of domestic violence (DV) during pregnancy has multiple detrimental effects on the mother and family, resulting in mental health impairment. In a cognizant effort to empower pregnant women who have experienced DV, a Behavioral Intervention Package (BIP) was developed and used. The BIP incorporates yoga-based techniques for self-development, interpersonal skill development, and awareness sessions. The study aims to assess the effect of a BIP on the quality of life (QOL), DV, anxiety, depression, PTSD and coping mechanisms of Indian women experiencing violence during pregnancy.

**Methods:**

This randomized controlled trial (RCT) involving pregnant women experiencing DV and attending antenatal care (ANC) was conducted at Lok Nayak (LN) Hospital in New Delhi. It is a tertiary care government hospital that provides free maternal healthcare services and primarily caters to individuals from low and middle socio-economic status. Based on the inclusion criteria, 921 participants were screened, and finally, 211 women were randomly assigned to either the intervention group (*n* = 105) or the control group (*n* = 106). The intervention consisted of two components: (A) a BIP and (B) standard care. The intervention group received both A and B, while the control group received only B. The BIP, delivered over 11 sessions, aimed to empower women to attain better physical and mental health. Over the seven-month period, each participant in both groups attended 11 sessions to receive the full intervention package.

**Results:**

The mean age of women in the intervention group was 25.3 years, and in the control group, it is 24.5 years. The intervention group showed significant improvements in QOL, with increases in physical (6.933 vs. −3.121) and mental health scores (7.802 vs. −3.623). Anxiety decreased (effect size 9.979), and there were significant reductions in depression scores (8.882), PTSD, and DV. Improvements in coping strategies (MD = 1.1, 95% CI = −1.47, −0.71) and social support (MD = 1.57) were also observed.

**Conclusion:**

The BIP can positively impact the mental health of pregnant women experiencing DV and attending ANC in India. As no standardized intervention exists for this population attending ANC in a hospital in India, integrating the BIP as an intervention during ANC is recommended.

**Clinical trial registration:**

Identifier, CTRI/2019/01/017009. https://ctri.nic.in/Clinicaltrials/advsearch.php.

## Introduction

Domestic violence (DV) is a ubiquitous public health issue affecting women in many ways worldwide (1, 2). The intersection of DV during pregnancy is known to lead to adverse health outcomes not only for the woman but also for the growing fetus, such as the risk of preterm birth and low birth weight due to the direct trauma or abuse and the physiological effects of stress on fetal growth and development (3, 4). Violence can have a negative impact on mental health of the mothers in several ways, including behavioral issues, (eating and sleeping disorders, self-harm and suicide attempts, and substance abuse) post-traumatic stress disorder (PTSD), depression, anxiety, and low self-esteem, (5, 6). Women who experience partner abuse during pregnancy have been found to have higher anxiety levels, even 6 months after childbirth (7). A longitudinal study documented that maternal antenatal stress could predict children’s behavioral and emotional issues up to 4 years later. Multiple detrimental effects on the mother, fetus, and family may result from mental health impairment (8). Neurobiological research shows that newborns exposed to in-utero domestic and family violence have elevated levels of stress-related hormones, including cortisol, which are linked to long-term effects (9, 10).

Behavioral interventions can significantly improve the mental health of pregnant women experiencing DV. These interventions typically involve a combination of counseling, support groups, safety planning, referrals to other services, and empowerment training (11, 12). A significant body of research supports universal screening for DV and sexual assault in healthcare and social support settings, such as antenatal care (13, 14). While universal screening has been shown to increase disclosures, there is limited evidence to suggest that it results in more referrals or reduces abuse (14). The MOVE model, developed for maternal and child health nurse screening of DV in Victoria, is a well-tested example (15). A randomized study in the United States demonstrated the effectiveness of a brief, evidence-based behavioral intervention for pregnant women disclosing DV in healthcare settings (16). Perinatal and postnatal parent-infant therapies can positively influence infants’ attachment and psychosocial development (17). Home visitation and peer mentoring programs during pregnancy and early parenthood may help reduce future DV among vulnerable families (18). The MOSAIC program, a home-visitation intervention for at-risk pregnant women and new mothers, aims to reduce DV and depression while strengthening mother–child bonding (19, 20).

An evaluation of current strategies intended at reducing the effect of intimate partner violence showed that individual-focused and couple-based interventions were significantly more effective in reducing violence, marital conflict, and forced sexual relations, with shorter durations (4–6 weeks) (21–23). In one Indian study, daughters-in-law (DIL) and mothers-in-law (MIL) met with an experienced counselor for 1 h at the clinic, with appointments scheduled outside regular hours if needed. Results showed that young women experiencing DV could use their MILs’ experiences as helpful resources in resolving conflict and dispute (24). Another intervention study targeting low-income African American pregnant women divided participants into intervention and routine care groups, with prenatal sessions addressing risks such as smoking, depression, and DV during routine antenatal visits (25). A study by Sapkota et al. reported that an intervention incorporating strategies like stress management, problem-solving, supportive counseling, empathetic listening, non-judgmental guidance, decision-making training, and facilitation significantly improved participants’ coping abilities and reduced symptoms of distress (26).

Despite the high prevalence of DV in India and a growing understanding of its determinants and detrimental health impacts, there is limited empirical evidence on effective DV interventions. Most interventions reviewed involve empowerment and related educational components, including emotional support, practical support, information and/or mediation. Other structural interventions have included providing legal aid in child custody cases, and support for women’s financial independence. However, little research has focused on behavioral interventions and coping strategies for pregnant women experiencing DV that preserve their psychological functioning and physical well-being. No standardized intervention exists for pregnant women experiencing DV and attending ANC in government hospitals in India. The BIP tailored for pregnant women, empowers them through yoga-based techniques for self-development, interpersonal skill-building, and awareness sessions, enhancing their agency in problem-solving and resilience. Therefore, this study aims to assess the effect of a BIP on the QOL, DV, anxiety, depression, PTSD, and coping mechanisms in Indian women experiencing violence during pregnancy.

A paper was published from the same research work focusing on the effect of BIP on the QOL, where a detailed analysis of QOL was provided, covering all 8 domains. These domains include physical function (PF—10 items), role physical (RP—4 items), bodily pain (BP—2 items), general health (GH—5 items), vitality (VT—4 items), social functioning (SF—2 items), role emotional (RE—3 items), and mental health (MH—5 items). Additionally, the paper examined the effect of BIP on reproductive and child health, as well as domestic violence (DV) severity (mild, moderate, and severe). This article, however, will analyze the QOL, which is scored using two composite scores: the Physical Component Summary (PCS) and the Mental Component Summary (MCS). Since the primary objective of the research is to assess QOL, it is important to provide a comprehensive overview of it. The changes in DV were analyzed based on the total score. The main focus of this paper is mental health, including anxiety, depression, and PTSD, as well as coping mechanisms.

## Methods

### Participants

**Figure 1:**
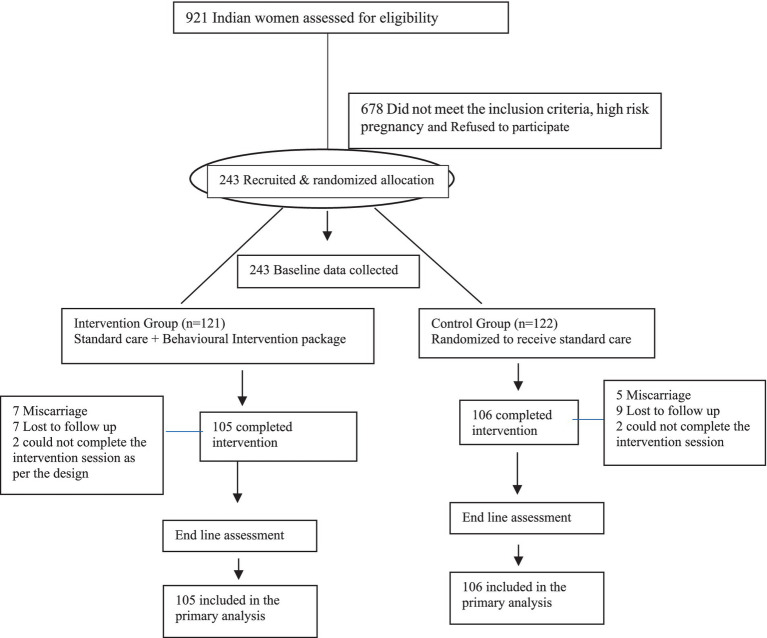
Flow of participants through the study. Reprinted with permission from Mahapatro et al. (28), licensed under CC BY 4.0.

Overall, 921 pregnant women were screened, of whom 678 also had DV but were not eligible for or excluded from the study. 243 women were randomized into either the intervention (*n* = 121) or control (*n* = 122) group. Thirty-two women (13.2%) dropped out of the study, therefore, 211 women entered the study and control groups, and data collection was completed for all 211 women (Figure 1). Most of them attended all the sessions of the intervention. There were no reports of adverse events or harm arising from participation in the study (Table 1) (28).

**Table 1:** Comparison of socio-demographic variable data in intervention and control groups.

Variables		Intervention group *N* = 105	Control group *N* = 106	*p*-value
Age (in years)		25.3(±)4.2	24.5 ± 3.6	0.14
Caste	General	36(34.3)	36(34.0)	0.487
	OBC	57(54.3)	60(56.6)	
	Others	12(11.4)	10(9.4)	
Religion	Hindu	34(32.4)	40(37.7)	0.999
	Muslim	71(67.6)	66(62.3)	
Women Education	Illiterate	8(7.6)	10(9.4)	0.318
	Primary	10(9.5)	17(16.0)	
	Middle	26(24.8)	16(15.1)	
	High-school	34(32.4)	44(41.5)	
	Above higher-secondary school	27(25.7)	19(17.9)	
Husband education	Illiterate	12(11.4)	12(11.3)	0.227
	Primary	8(7.6)	11(10.4)	
	Middle	29(27.6)	27(25.5)	
	High-school	33(31.4)	32(30.2)	
	Above higher-secondary school	23(21.9)	24(22.6)	
Women occupation	House-wife	100(95.2)	101(95.3)	0.999
	Working	5(4.8)	5(4.7)	
Husband occupation	Organized sector	68(64.8)	58(54.7)	0.460
	Self-employed	15(14.3)	30(28.3)	
	Unemployed	22(21.0)	18(17.0)	
Type of family	Nuclear	31(29.5)	14(13.2)	0.754
	Joint	74(70.5)	92(86.8)	
Total family income (in Rs per month)		20599.0(±)23795.2	22845.3(±)28706.6	0.545

Values are expressed as mean ± SD or frequency (%).

### Inclusion and exclusion criteria

The inclusion criteria comprised of married pregnant women (between 18 and 20 weeks of pregnancy) attending the hospital’s obstetrics outpatient department (OPD) for antenatal care, who had been screened positive for DV during the past year using the Abuse Assessment Screening Tool (AAST) (27). Additionally, participants needed to be living with their husband or in-laws (family) for at least 2 years, likely to continue treatment until delivery, and willing to attend follow-up appointments at the hospital as scheduled. Informed written consent was obtained from all participants. Women excluded from this study were those already registered as medico-legal cases (MLCs) related to abuse, and those with intellectual disabilities that affected their ability to comprehend and comply with the intervention. They were excluded at the screening level.

### Trial design and setting

This RCT among pregnant women experiencing DV was conducted from November 2018 to June 2020 at a tertiary care, LN hospital in New Delhi. The LN hospital is a government hospital that provides free services and caters to people of low and middle socio-economic status. A designated space with adequate seclusion was created in the OPD area of the hospital where women could speak freely without being overheard by their accompanied relatives. The pregnant women were approached by the women researchers in the OPD area. The interviews were conducted in the participants’ native language by the women researchers.

### Data collection

- Quality of life (QOL) (28): The SF-36 comprises two composite scores (The eight domains are summarized into two composite scores)—the Physical Component Summary (PCS) and the Mental Component Summary (MCS), each ranging from 0 to 100, with higher scores indicating better health. The Cronbach’s alpha for this tool is 0.399.
- Abuse Assessment Screening Tool (AAST) (27): This tool contains 15 items developed and validated in Indian cultural context. It assesses the type, frequency, duration, abuser-relationship with the abuser, and severity of violence. Responses are scored as either ‘yes’ or ‘no.’ The Cronbach’s alpha for this tool is 0.989.
- The Hamilton Anxiety Rating Scale (HAM-A) (29): It consists of 14 symptom-defined elements and measures psycho-social and somatic symptoms such as tension, fear, insomnia, sensory, respiratory, gastrointestinal, cardiovascular, etc. Each item is rated in a five-point score, from 0 to 4, where 0 is ‘not present’, 1 is ‘mild’, 2 is ‘moderate’, 3 is ‘severe,’ and 4 is ‘very severe.’ The total score is the summation of all individual item scores, and >17/56 is taken to indicate mild anxiety; 25–30 is considered moderate–severe anxiety. The Cronbach’s alpha for this tool is 0.879.
- Hamilton Depression Rating Scale (HDRS) (30): A 17-item scale, measures the severity of depressive symptoms by probing into the mood, feelings of guilt, insomnia, anxiety, weight loss, etc. Each item on the questionnaire is scored on a 3 or 5-point scale based on the item. The individual items are scored individually, and the total scoring is based on the 17-item scale. The scores of 0–7 are considered normal, 8–16 suggest mild depression, 17–23 moderate depression, and scores over 24 indicate severe depression. The maximum score is 52 on the 17-point scale. The Cronbach’s alpha for this tool is 0.804.
- Post-traumatic stress disorder (PTSD-8) (31): It is a short screening tool derived from the first 16 items of the Harvard Trauma Questionnaire Part IV (HTQ), which corresponds to the Diagnostic and Statistical Manual of Mental Disorders (DSM-IV) criteria for PTSD. The 8 items are answered on a four-point Likert scale where 1 is ‘not at all,’ 2 is a little, 3 is ‘quite a bit,’ and 4 is ‘all the time’. The summed-up score provides a score for symptom severity. The eight items relate to intrusive recollection, event recurring, recurrent dreams, psychological and physiological distress, efforts to avoid activities, thoughts, exaggerated startled response, and hypervigilance. The Cronbach’s alpha for this tool is 0.83.
- Coping Checklist (32): It is a 14-item scale under five subscales; problem-focused (3 items), seeking social support (4 items), avoidance (5 items), and collusion (1 items), coercion (1 item). Each item is scored on a three-point scale, where 1 is ‘never’, 2 is ‘sometimes,’ and 3 is ‘always’. The Cronbach’s alpha for this tool is 0.683.

### Intervention

#### Standard care

The standard of care focused on the routine services provided by health professionals at the antenatal clinic, which include ANC, natal care (NC), postnatal care (PNC), and child care. It also encompasses health promotion and well-being through education and support in areas such as nutrition, substance abuse cessation, family planning, recognition of danger signs, and birth preparedness.

#### BIP

The BIP was developed based on the review of different existing national and international guidelines (33), which are context specific. The intervention comprised a BIP addressing the complex life situation and the key challenges faced by women, such as making strategic life choices, having access to resources, acknowledging their achievements, and enabling them to exercise agency. This intervention aims to empower women to attain better physical and mental health. BIP consisted of five components, while the standard care focused on the routine services provided by health professionals at the antenatal clinic. These five BIP components were focused on i. Understanding the depth of the problem and assessing the need with empathy and rapport; ii. Analyzing her strengths and available resources (emotional, medical, and physical resources) for utilization and navigating a better outcome; iii. Self-regulation mechanisms of the body’s internal system through yoga-based methods (chanting, meditation, and exercise); iv. Individual counseling for effective communication and better interpersonal relations; and v. Developing better awareness and creating opportunities for alternative livelihoods.

### Study outcomes

The primary outcome of the study is QOL, measured using the SF-36. The secondary outcomes include: i. reduction in anxiety (HAM-A), ii. depression (HDRS), iii. PTSD (PTSD-8), iv. changes in the recurrence of DV (AAST) v. changes in coping mechanisms (coping checklist). All the outcomes were measured at baseline and post-intervention.

### Sample size

In the previous study by Tiwari and colleagues (12), general health was used as the outcome indicator for calculating the sample size. Assuming that the general health is 53 ± 7.5 in the study group and 50 ± 7 in the control group, a minor change is expected. Hypothesis testing for two means is based on the t-test formula with following assumptions.

- The outcome variable is continuous.
- The sampling distribution of the sample mean is approximately normal.
- The observations are independent.
- The variances in the two groups are similar.

Formula

$n=\frac{2S_{p}^{2}{\left[Z_{1-\alpha /2}+Z_{\beta }\right]}^{2}}{\mu d^{2}}$

$S_{p}^{2}=\frac{S_{1}^{2}+S_{2}^{2}}{2}$

Where,

S_1_^2^: Standard deviation in the first group.

S_2_^2^: Standard deviation in the first group.

μ_d_^2^: Mean difference between the samples.

*α*: Significance level.

1 – *β*: Power.

Two means: Hypothesis testing for two means.

**Table:** 

Items	Col. 1
Standard deviation in group I	7.5
Standard deviation in group II	7
Mean difference	3
Effect size	0.413793
α Error [%]	5
Power [1 – β] %	80
1 or 2 sided	2
Required sample size per group	92 + 92 = 184

Further, assuming α (type I error) of 5% and power taken as 80%, a sample of 184 cases was required to be enrolled. It was rounded up to hundreds to achieve a figure of 200. Taking into account a 20% non-compliance rate, as well as the likelihood of dropouts due to genetic markers, probability of abortion and culture-specific reasons for dropouts, the required sample size was adjusted to 220.

### Recruitment and consent

Pregnant women who met the eligibility criteria were screened using the ASST tool, and those who answered “yes” regarding abuse within the last year of the study were considered and enrolled. The participants were provided with an explanation of the study’s purpose, potential risks and benefits, instruments, administration time, and follow-up schedules, and were given the subject information sheet for consent. If a participant agreed to participate, written informed consent was obtained, and the participant was enrolled in the study.

### Randomization and blinding

Eligible participants were randomized to either the intervention or the control group at a 1:1 ratio. A computer-generated list sequence was concealed in consecutively numbered, sealed envelopes and recorded by an investigator who was not involved in the study. The research assistants who entered and analyzed the data were recruited after data collection in the third phase and, therefore, did not know the study hypotheses or design and were blinded to group assignment. The person administering the intervention and standard care packages differed for the groups and were not interchangeable.

### Research procedure

The BIP and standard care were administered to each woman in the intervention group, while the control group did not receive the BIP and only received standard care. Standard care was provided to all women in both groups. The focus of the interactions and discussions was on the healthy development and well-being of both the mother and the fetus. Although this is part of routine care, the research team emphasized and personalized this information as part of individual-centered care.

The intervention was administered by senior researchers trained in clinical psychology, community medicine, gynecology, anthropology, and yoga. Each one-to-one session, which was conducted in a private room near the OPD, lasted about 45–70 min and took place without the male partner or other family members present. Over a period of 28 weeks, each woman received 11 sessions to complete the entire intervention package. The intervention was administered over 7 months, including up to 6 weeks postnatal. The control group received the standard care, which also consisted of 11 sessions with the researcher, similar to the intervention group (28).

### Data analysis

Statistical analysis was performed using SPSS 20 software. The data are presented as frequencies and percentages. Continuous data are expressed as either the mean ±SD or median (inter-quartile range or IQR) as appropriate. A student t-test was applied to compare continuous data between the groups, measured by the McNemar test, and a Chi-square test was applied to compare categorical data. The QOL, AAST, Coping Checklist, PTSD-8, HDRS, and HAM-A, along with their subscales were compared pre- and post-intervention using a paired t-test, or Wilcoxon signed-rank test, as appropriate to estimate the effect size at baseline and post-intervention. Effect size (Cohen’s d) was calculated at baseline and post-intervention. Additionally, 95% confidence intervals (CI) were calculated. Of the 243 women who initially entered the study, baseline data were collected for all, and 211 women completed the intervention. Therefore, the analysis was conducted on the 211 participants who completed the study.

## Results

### The preliminary analysis

The mean (SD) of the women’s age in the intervention group was 25.3 (3.6) years, and in the control group, it was 24.5 (3.6) years. About 18% of the women in the intervention group and 25% in the control group had 12 years of schooling or more. Women were predominantly housewives (95%) and belonged to a lower economic category. The participants in both groups mostly belonged to the general caste category (about 34%) and (OBC) (about 55%). More women were living in joint families in the control group (about 87%) than in the intervention group (about 70%). At baseline, the intervention and control groups were comparable in all socio-economic characteristics (Table 1).

### Intervention effect on QOL

The primary outcome, QOL, includes two domains: Physical Component Summary (PCS) and Mental Component Summary (MCS) (Table 2). At baseline, the groups were similar concerning the QOL and its components [PCS and MCS = intervention group 38.7(±)5.2 & 37.6(±)5.9, control group 37.0(±)4.8 & 37.5(±)4.9 respectively]. Post-intervention, there were significant mean changes in the intervention group in QOL subscales for both domains (*p* < 0.001) compared to the baseline. After 28 weeks, the between-group difference indicated a statistically significant increase in the QOL in the intervention group in comparison to the control group: physical health (6.933 vs. −3.121) and mental health (7.802 vs. −3.623) respectively.

**Table 2:** Comparison of quality of life (QoL) between intervention and control group, pre and post-intervention.

QoL		Intervention group *N* = 105 (mean ± S.D)	Control group *N* = 106 (mean ± S.D.)	Effect size (95% C.I.)	*p*-value
Physical Component Summary (PCS)	Pre	38.7(±)5.2	37.0(±)4.8	1.779 (0.410, 3.149)	0.011
	Post	45.6(±)8.0	33.8(±)5.9	11.834 (9.918, 13.749)	<0.001
	Mean difference between baseline & endline	6.933	-3.121		
	Effect Size	−0.78 (−0.9, −0.56)	0.43 (0.23, 0.63)		
	*p*-value	<0.001	<0.001		
Mental Component Summary (MCS)	Pre	37.6(±)5.9	37.5(±)4.9	0.137 (−1.327, 1.601)	0.854
	Post	45.4(±)7.1	33.9(±)7.7	11.562 (9.553, 13.572)	<0.001
	Mean difference between baseline & endline	7.802	−3.623		
	Effect Size	−0.90 (−1.13, −0.67)	0.41 (0.21, 0.61)		
	*p*-value	<0.001	<0.001		

### The intervention effects on violence

The mean difference of the score in the control group is 0.708, while in the intervention group, it is 1.962 (Table 3). The baseline values were comparable between the control and intervention groups (*p* = 0.98), whereas, after the intervention, there was a difference between the two groups. Further, there was a significant decrease in DV in the intervention group compared to the control group, and the change in the intervention group was observed as 1.96 ± 2.29, significantly higher than the control group (*p* = <0.001).

**Table 3:** Comparison of domestic violence experience between intervention and control group, pre and post intervention.

Outcome variable	Pre-intervention	Post-intervention	Two-sided *p*
Control *N* = 106 (mean ± S.D.)	3.46(±)2.431	2.75(±)2.309	<0.001
Intervention *N* = 105 (mean ± S.D)	3.50(±)2.366	1.54(±)1.676	<0.001

### Intervention effect on anxiety and depression

The intervention effect shows a significant improvement in reducing anxiety in the intervention group with a 9.979 (95% CI = 7.71, 12.24) score even after adjusting baseline anxiety as the intervention group mean anxiety score was 11.18 which was significantly (*p* < 0.001) less than the control group anxiety score. On the regression line, the equation for anxiety score was = 15.36–11.18xstatus (1 if intervention group, and 0 if control group) + 0.33x anxiety score at baseline. It has a correlation, and the variance explained by the model R2 was observed as 0.35. A similar observation was found with a significant lower depression score in intervention group 8.882 (95% CI = 7.33, 10.43); even after adjusting the baseline depression score, the mean depression score was 9.78, which was less than the control depression score and was statistically significant (*p* < 0.001). On the regression line, the equation for depression score was = 12.38–9.783xstatus + 0.278x depression score at the baseline had having correlation, and the variance explained by the model R2 was observed as 0.44. Assuming that these (pre and post-test) were independent (Table 4).

**Table 4:** Comparison of anxiety, and depression score between study and control group, pre and post-intervention.

Outcome variables		Control group *N* = 106 (mean ± S.D.)	Intervention group *N* = 105 (mean ± S.D)	Effect size mean differences (95% C.I.)	*p*-value
Anxiety	Pre	26.9(±)8.1	30.6(±)9.3	−3.7 (−6.043,-1.289)	0.003
	Post	24.1(±)8.8	14.2(±)7.8	9.98 (7.712,12.247)	<0.001
	Effect Size	(0.798,4.692)	(14.553,18.228)		
	*p*-value	0.006	<0.001		
Depression	Pre	18.1(±)5.8	21.3(±)7.3	−3.24 (−5.034,-1.445)	<0.001
	Post	17.4(±)6.7	8.5(±)4.4	8.882 (7.333,10.430)	<0.001
	Effect Size	(−0.760,2.193)	(11.540,14.136)		
	*p*-value	0.338	<0.001		

### Intervention effect on PTSD

There was a significant change with *p*-value <0.05 in the pre and post-value in all domains of the intervention group, except in one domain: efforts to avoid thoughts. Most of the domain was found to be non-significant at the baseline except for psychological and physiological distress and, therefore, comparable. At the baseline and end-line, the difference between the total mean score and SD is 4.90 + 6.26 and 1.37 + 6.72, respectively. Delta change in the intervention group is 4 times that of the control group, as there is a 20% change in the intervention group to a 5.7% change in the control group (Table 5).

**Table 5:** Comparison of PTSD score between study and control group, pre and post-intervention.

Domain	Group	N	Paired *t*-test (mean & SD)		*p* value
			Baseline	Endline	
Intrusive recollection	Intervention	105	3.38 ± 0.801	2.62 ± 0.944	0.000
	Control	106	3.30 ± 0.886	2.92 ± 1.011	0.002
Event recurring	Intervention	105	3.27 ± 1.049	2.52 ± 1.001	0.000
	Control	106	3.27 ± 0.889	2.89 ± 1.072	0.001
Recurrent dreams	Intervention	105	2.49 ± 1.178	1.71 ± 0.927	0.000
	Control	106	2.57 ± 1.155	2.39 ± 1.092	0.224
Psychological and physiological distress	Intervention	105	3.45 ± 0.808	2.59 ± 0.968	0.000
	Control	106	3.18 ± 0.934	2.99 ± 0.981	0.126
Efforts to avoid activities	Intervention	105	3.15 ± 0.928	2.78 ± 1.118	0.007
	Control	106	2.97 ± 0.833	3.10 ± 0.965	0.224
Efforts to avoid thoughts	Intervention	105	3.10 ± 0.986	2.89 ± 1.077	0.110
	Control	106	3.08 ± 0.870	2.98 ± 1.014	0.467
Exaggerated startle response	Intervention	105	3.14 ± 0.975	2.39 ± 0.946	0.000
	Control	106	3.08 ± 0.927	2.94 ± 0.994	0.197
Hypervigilance	Intervention	105	3.13 ± 0.971	2.70 ± 1.011	0.000
	Control	106	3.20 ± 0.899	3.06 ± 0.934	0.235
Total Difference	Intervention	105	25.10 ± 5.125	20.20 ± 5.668	0.001
	Control	106	24.65 ± 5.15	23.27 ± 5.696	0.037

### Intervention effect on coping

Results of post-intervention coping score indicated effects on improvement in problem-focused approach (MD = 1.1, 95% CI = −1.47, −0.71) and social support (MD = 1.57, 95% CI = −2.11, −1.03), which were statistically significant (*p* < 0.001) in the intervention group. The intervention group also showed a significant increase of 12.7% in the problem-focused approach and 21% in social support, respectively. Though it is not statistically significant, there was a trend toward a decrease in avoidance and collusion coping in the intervention group (Table 6). The control group did not improve problem-focused coping, and there was an increase in avoidance and collusion coping.

**Table 6:** Comparison of coping score between study and control group, pre and post-intervention.

Outcome variables		Control group *N* = 106 (mean ± SD)	Intervention group *N* = 105 (mean ± SD)	Effect size mean differences (95% C.I.)	*p*-value
Problem focused	Pre	5.9(±)1.5	6.3(±)1.7	−0.456 (−0.89,-0.02)	0.04
	Post	6.0(±)1.4	7.1(±)1.3	−1.096 (−1.47,-0.71)	<0.001
	Effect Size	(−0.46,0.13)	(−1.14,-0.45)		
	*p*-value	0.291	<0.001		
Social Support	Pre	6.2(±)1.6	7.0(±)2.0	−0.821 (−1.32,-0.31)	0.002
	Post	6.9(±)1.9	8.5(±)2.0	−1.57 (−2.11,-1.03)	<0.001
	Effect Size	(−1.10,-0.36)	(−1.93,-1.03)		
	*p*-value	<0.001	<0.001		
Avoidance	Pre	7.6(±)1.5	8.3(±)1.8	−0.691 (−1.16,-0.22)	0.004
	Post	8.0(±)1.5	8.0(±)1.6	0.027 (−0.39,0.45)	0.899
	Effect Size	(−0.81,-0.01)	(−0.16,0.74)		
	*p*-value	0.044	0.210		
Collusion/ Coercion	Pre	3.9(±)1.1	4.0(±)1.2	−0.180 (−0.50,0.14)	0.273
	Post	4.0(±)0.9	3.8(±)0.9	0.115 (−0.15,0.38)	0.396
	Effect size	(−0.34,0.15)	(−0.06,0.45)		
	*p*-value	0.464	0.143		

## Discussion

Overall, the study showed the efficacy of BIP given to pregnant women experiencing DV during their ANC in a clinical setting in India. The intervention led to improvements in women’s psychosocial status, mental well-being, and coping mechanisms. The intervention significantly reduced anxiety in the intervention group, even after adjusting for baseline anxiety. A similar reduction in depression scores was observed, with the intervention group showing significantly lower depression scores than the control group. There was a significant change (*p*-value <0.05) in all PTSD domains in the intervention group, except for “efforts to avoid thoughts.” The delta change in the intervention group was four times greater than in the control group, with a 20% change in the intervention group compared to a 5.7% change in the control group. These results align with studies from other Asian countries and globally (34, 35). Women who underwent this intervention could work on regulating their internal systems of the body, emotions, and thoughts with the help of yoga-based and techniques. Additionally, simple group activities and discussion helped to improve their basic hygiene, self-care and thus enhancing self-perception.

The results indicated that post-intervention, the BIP had effects on coping mechanisms employed by the women. However, there was limited change in avoidance and collusion, suggesting that a longer, more extensive intervention may be needed. Over time, due to BIP interventions, the women were able to start thinking about the actual problem and potential solutions, while also increasing their perceived social support. However, since they continued to live in the same households within the families, where the abuse had been taking place, and the process of resolution was still ongoing, avoidance and collusion were likely used as ways of averting the potential escalation of conflict. As they became more oriented toward problem-solving and developing the social support systems they needed, they sometimes resorted to avoidance and collusion for self-protection. Our data indicated that, overall, the trajectory of recovery was unfolding in the right direction. This was reflected in the reduction of depression and PTSD scores. PTSD thrives on unresolved trauma, and the anxiety keeps recurring and the thought patterns associated with the violence and abuse are recalled back in the memory. However, with an increased ability to start solving the problem, address the situation, and gather social support, the women were able to gradually overcome and start managing their anxiety and depression, which had previously dominated their lives.

Additionally, the intervention improved the control group’s social support coping from baseline to post-intervention. This could be attributed to the researcher’s interaction with the women and the provision of respectful standard care, which is often not expected in hospital settings. The analysis highlights the adaptive nature of coping strategies, allowing women experiencing DV to determine how best to respond to their circumstances. In comparison, the control group did not show improvement in problem-focused coping; on the other hand, avoidance increased. Consistent with previous RCTs, mind–body interventions have proven effective in reducing DV, anxiety, and depression while enhancing coping mechanisms in women (28, 36, 37). Addressing the psychological and emotional aspects of their experiences along with providing support, can help mitigate the harmful effects of violence, improve mental well-being, and promote healthier outcomes for both women and their unborn children. The comprehensive intervention improves or raises their awareness, coping mechanisms, and emotional responses to violence, and can be integrated into existing healthcare delivery systems (12, 38).

## Strengths and limitations

To our knowledge, this is the first RCT specifically examining the effects of a BIP intervention on the mental health and coping mechanisms of pregnant women who have experienced DV in India. The researcher observed high retention rates and compliance with the intervention, which was consistent with previous trials involving women who have experienced abuse (12). The research also demonstrated the utility and feasibility of employing an innovative, integrated approach.

This study has several limitations. All measures were self-reported, making them susceptible to subjective errors (35). Additionally, women’s initial responses may differ from post-intervention responses due to the increased trust developed over the course of multiple sessions. Another limitation is the focus on women’s coping efforts with DV without considering the involvement of their partners or families in the therapeutic process. The lack of a thorough understanding of the perpetrator’s actions—due to factors such as attitude, values, psychopathology, personality disorders, and socio-cultural conditioning—also limits the study (34, 39). Finally, the intervention’s effects have primarily been applied to low socio-economic women attending ANC at a public hospital, so testing it in other socio-demographic groups would be important to assess the generalizability of the results.

## Conclusion

Overall, behavioral interventions can make a positive difference in the mental health of pregnant women experiencing DV in India. The BIP approach is a deliberate effort to address anxiety, depression, and PTSD of pregnant women experiencing DV. Currently, there is no standardized intervention package in India that specifically addresses the psychosocial health needs of women experiencing violence. There is a need for a flexible intervention tool that can be adapted to the diverse situations and settings of women presenting with DV. It is important that such an intervention takes a holistic approach, addressing physical, mental, and psychosocial health while also incorporating preventive measures. Since screening tools are not yet routinely used, the BIP could serve as both a screening and intervention package, integrated into ANC to improve accessibility and outcomes.

## Data Availability

The raw data supporting the conclusions of this article will be made available by the authors, without undue reservation.
